# An Account of the Bilious Remittent Fever of 1828; as It Prevailed in Circleville, and Its Vicinity; and, Especially, along the Line of the Ohio and Erie Canal, near That Town

**Published:** 1829

**Authors:** John Cook Bennett

**Affiliations:** Circleville, Ohio


ART. II.—*An account of the Bilious Remittent Fever of* 1828; *as it prevailed in Circleville, and its vicinity; and, especially, along the line of the Ohio and Erie canal, near that town.* By DR. JOHN COOK BENNETT.

### DURATION AND EXTENT OF THE EPIDEMIC.

In the country, it commenced about the middle of August (1828) and continued to the middle of October. In Circleville, it prevailed but a fortnight. It affected the inhabitants, generally, throughout that section of the valley of the Scioto in which the town is situated; but its chief prevalence was along the line of the canal, where a great number of labourers were engaged in the work of excavation. Among these it was so severe, as to cause a general panic; and, in many places, an entire suspension of labour, for several weeks.

### REMOTE CAUSES.

The wind during the period to which I have referred, was variable, and sometimes vehement. The proportion of cloudy weather was considerable; but although showers were frequent, some parts of the season were too dry for active vegetation. The atmosphere at times was extremely humid, at others, hot and sultry.

In the neighbourhood of Circleville, there are ponds and milldams; and immediately about the town a moat, formed by two circular, concentric, embankments—a part of the antiquities of the Western country.* The whole of these collections of stagnant water were covered with a green pellicle; and to the decomposition of vegetable matter, of which they were the seats, we may, I think, refer the fever which occurred in their vicinity. But no such collections of stagnant water existed where the canal excavations were

---

*See Archæologia Americana.

carried on; notwithstanding which, the fever was there most prevalent and most fatal. Still, its cause, I conceive, was the same. The excavation was through alluvial soil, thoroughly impregnated with vegetable matter, long buried beneath the surface; and to the exhalations from this matter, exposed to the action of the rains, sun and air, we are justified in ascribing the extension of the epidemic to that quarter. We may even conjecture, that the miasms arising from the recently disenterred vegetable recrements, had a peculiar degree of virulence; since the most fatal cases occurred in their neighbourhood. The canal labourers, however, were subjected to the influence of several auxiliary or predisposing causes, which did not operate on the resident population of either town or country:—1. They were badly lodged, in temporary huts, cabins, and shanties; most of which had earthen floors;—2. They were greatly exposed to the heat of the sun, and to rain;—3. Their diet was frequently of an improper kind;—4. Some of them were strangers to the climate, and others unaccustomed to the kind of life which they then pursued:—But that the exhalations from the newly broken alluvial soil, were the chief cause, was rendered almost certain, by the fact, that the resident inhabitants of the country along the line of the canal, were more severely affected than others, living at a greater distance.

### Symptoms and Progress of the Fever.

The *cold stage* varied in duration, from one to six hours, with a small and frequent pulse. In general there was excessive pain in the head, back and loins. The respiration was laborious, with great oppression in the precordia. The *Hot stage* brought out high toned inflammatory symptoms. It was attended with burning heat over the whole surface, but especially the epigastric region. The pulse, retaining its frequency, became full and hard. The pains of the head and spine were greatly aggravated; but the sense of oppression in the precordia was diminished. The laborious respiration continued. The eyes acquired a heavy aspect; and the face a flush. The temporal arteries throbbed excessively. The limbs were affected with pain and lassitude. The thirst was excessive. At an early period of the disease, the tongue became dry, rough, and discoloured. When the malady tended to a fatal termination, coma supervened, the præcordial oppression increased, the respiration became more difficult, the face swelled, the muscles became tremulous and even convulsed, the skin, in many cases, assumed a bright yellow colour, and the cold sweats, which preceded death, stained the shirts of a saffron colour.

In cases of a more favorable character, the paroxysms varied in their duration from 12 to 36 hours; when a gentle perspiration, attended with a mitigation of all the symptoms, would break out. The disease was not prone, however, to assume the intermitting type, and in most cases there was only a remission, and this frequently of short duration.

TREATMENT.

"In fevers," says Dr. Miner, "the physician who only employs a palliative treatment, resembles the temporising, nerveless politician, who endeavours to repel foreign aggression, by bribing the enemy or paying tribute; but he that combats disease by making an early and powerful impression, is the efficient general, who transfers the seat of war into the enemy's country." This decided plan of an 'early and powerful impression,' was pursued in Circleville and its vicinity, not only by myself, but by Drs. Gibson, Luckey, Noble, Webb, and Hill, all respectable physicians, and proved to be the only one on which we could rest with any degree of safety. The same plan was pursued by Drs. Hildreth, Cotton, German and myself, in the fever of 1822—3, at Marietta, an account of which was published by the first named gentleman, in the Philadelphia Journal. Dr. Regnier of Galliopolis, Dr. Johnson of McConnelsville, Dr. Appleck of Barnesville, and Dr. Ackly, a former student of mine, have all pursued, successfully, the same course.

When called to treat a patient labouring under this disease, I commenced with blood letting, unless he had been ill for several days. The quantity taken varied from one to three pints, and the effects were eminently beneficial. Immediately after the operation, I began the exhibition of calomel, in *forty-grain-doses*, every eight hours, and continued it for two or three days, during which the contents of the biliary ducts and the bowels, were freely evacuated. The dose of calomel was then reduced to two grains and repeated every two or three hours, until ptyalism occurred. If too much purging resulted from this administration, which often happened, I combined opium with the medicine, in such quantities, as to restrain the discharges to one or two each day. In some cases, however, in which the calomel, either in small or large doses, failed to produce sufficient purging, I found castor oil highly beneficial. Thus blood-letting and calomel were the great means of cure. The former prepared the system for the latter. Neither could have succeeded alone. I saw no case of death after the occurrence of a salivation. As the loss of blood in most cases, brought the excitement of the system to the grade which favoured the action of the calomel; so, on the other hand, when the excitement was too low, it was raised by stimulants to the degree which was necessary to the effect of that medicine.

In cases attended with obstinate constipation, injections were useful. Ipecacuanha given so as to vomit, after venesection, proved serviceable in some cases; and diaphoretics were also beneficial, but their effects were temporary.

Throughout the hot stage, I directed a free application of cold water to the surface of the body generally, and observed it to be attended with the happiest effects. It seemed to facilitate the action of the calomel on the mouth.

Epispastics to the forehead and extremities, about the fifth day, or as soon as the mercurial action had taken place, were of great advantage. If applied before the ptyalism had commenced, they prevented or retarded its establishment. On this point I could not be mistaken. If resorted to after the salivation had become violent, they never failed to check it. Indeed so great was their power in this respect, that I found them a valuable resource in all cases where the mercurial action threatened to become excessive. In accordance with these facts, I have observed for several years, in other diseases, that it is extremely difficult to salivate a patient, who is under the influence of blisters.*

When typhoid symptoms supervened, we resorted to stimulants, both diffusible and permanent. Among the latter, a decoction of equal parts of the bark of the *cornus florida*, or dog-wood, and the *prunus virginiana*, or wild cherry tree, rendered stimulating with whiskey, was attended with excellent effects.

The saline mixture (solution of pearlash in vinegar) was resorted to in some cases, but I am quite convinced, that from its being often prepared with an excess of alkali, it was in some instances highly injurious. I have even thought, that it sometimes eroded the mucous membrane of the stomach. Previously to the epidemic, in 1825, I saw it given to a patient, who discharged a considerable quantity of blood from the mouth immediately after swallowing it; and subsequently on taking calomel, he evacuated many fragments of flaky and membranous matter, resembling the mucous lining of the canal. In consequence of these effects, I seldom employed this mixture in the epidemic of 1828.

But the abuses of another medicine were much more dangerous. I allude to tartar emetic, which, as far as my observation extended, was always pernicious, in the fever under consideration. It seldom failed to excite in the stomach and bowels an ungovernable peristatic action which greatly exhausted the patient. Indeed, my previous experience with this medicine, had by no means prepossessed me in its favour. I had witnessed too many instances of it sinister operation not to doubt its safe and salutary effects. A fatal case of its abuse, fell under my notice in 1825. On the 3d of August, I was called in consultation with Dr. C. F. Perkins, to visit Miss H. G. labouring under the effects of a dose of tartar emetic, administered about four hours before. She lay speechless and senseless, except, to give evidence of excru-

*We take this opportunity to bear the testimony of our own experience, to the great efficacy of blisters, especially when applied to the back of the neck, in arresting the mucous, glandular and cellular inflammation of the mouth and throat, which sometimes occur under the administration of calomel. Editor ciating pain in the abdominal region. The alvine discharges were frequent and involuntary. Her eyes were turned upwards, her mouth extended, and her countenance ghastly. The thorax heaved high, and at length spasms with great distortion of features supervened, a dreadful gasping for breath came on, and in 16 or 18 hours after the medicine was taken, she expired.

Opium was given liberally, and checked the alvine evacuations, but afforded no other relief. Diffusible stimuli, both internal and external, with blisters to the head and extremities produced no effect.

But to return to our epidemic. The plan of treatment which I have detailed, was, in my own practice, so eminently successful, that I lost but one patient, and he expired the next day after I was called to see him, the 12th of his illness.

### Sequelæ.

The disease was succeeded by Jaundice, which affected a great number, and yielded to the ordinary remedies. Many cases of ague likewise followed, and assumed, generally, a quartan type—in one person the quintan. They were cured by the following method. I first bled to the extent of one or two pints; then administered a large dose of sulphate of magnesia, or calomel and jalap; after which I gave twelve drops of Fowler's solution of the arseniate of potash, five times a day, until the disease was removed, or the face began to swell. In some cases it was necessary to repeat the blood letting and purging, and to vary the dose of the solution. This plan did not fail in a single case.

### Non-Contagion.

When the disease was extremely prevalent along the canal, I had several persons, severely ill, brought into Circleville, at that time quite healthy, and not one of them communicated the disease to those about them;—a sufficient evidence that it was not contagious.

Circleville, Ohio, January 1829.

